# Navigating Through the Course of Autoimmune Hepatitis in Adults: A Case-Series Exploration

**DOI:** 10.7759/cureus.75152

**Published:** 2024-12-05

**Authors:** Onkar Awadhiya, Bharatsing D Rathod, Rajashree S Khot, Sachin R Chaudhari, Nilesh Kamble

**Affiliations:** 1 General Medicine, All India Institute of Medical Sciences, Nagpur, Nagpur, IND; 2 Pathology, All India Institute of Medical Sciences, Nagpur, Nagpur, IND

**Keywords:** acute hepatitis, autoimmune hepatitis (aih), autoimmunity, non-alcoholic cirrhosis, tuberculous peritonitis with cirrhosis

## Abstract

Autoimmune hepatitis (AIH) is a distinct clinical entity with variable presentations and diverse clinical outcomes, characterized by autoimmune-mediated injury to the liver. The detection of autoantibodies and histological features consistent with autoimmune injury is crucial for diagnosing AIH. Early identification and treatment are essential to prevent progression to cirrhosis.

This report describes five cases of AIH diagnosed and managed over one year, with presentations ranging from acute hepatitis to end-stage liver disease. Younger patients demonstrated good clinical and biochemical responses to immunosuppressive therapy, while an older patient with established cirrhosis faced a grim prognosis. AIH should be considered, particularly in female patients and in those without obvious risk factors for liver injury, such as alcohol use, drug-induced liver injury, or viral hepatitis.

## Introduction

Autoimmune hepatitis (AIH) is an immune-mediated liver disease characterized by transaminitis, hypergammaglobulinemia, autoantibodies, and interface hepatitis. It can affect people of all ages, from infants to older adults. Patients with AIH present with varied manifestations, ranging from incidentally detected asymptomatic transaminitis to acute fulminant liver failure. There is no single diagnostic marker for AIH. Diagnosis is made by excluding common etiologies of liver disease, detecting autoantibodies, and identifying characteristic histological features [1]. Two types of AIH are recognized, type 1 and type 2, based on the autoantibodies present. The disease may progress to advanced hepatic fibrosis, cirrhosis, or liver failure. Concomitant autoimmune conditions, such as autoimmune thyroid disease, vitiligo, rheumatoid arthritis, systemic lupus erythematosus, and type 1 diabetes mellitus, may also occur [2]. Treatment involves immunosuppression to curb immune-mediated progressive hepatic injury. First-line therapy includes steroids with or without azathioprine. In cases where remission is not achieved, or adverse effects of first-line therapy are significant, second-line treatment is considered. Treatment may have variable responses and their adverse effects are important considerations for the treatment. Pertinent issues in the management of AIH are timely diagnosis, appropriate immunosuppression administration, and treatment adherence. A liver transplant is a treatment option for AIH-associated cirrhosis.

AIH is considered rare in India. A hospital-based retrospective cohort study reported an AIH prevalence of 1.3%, with a prevalence of 8.74% among chronic liver disease patients [3]. In this case series, we describe five cases of AIH in adult patients admitted between January 2023 and December 2023 at a tertiary care hospital in central India. These cases highlight the heterogeneity in the clinical, biochemical, and immunological features of AIH.

## Case presentation

Case 1

**Figure 1:**
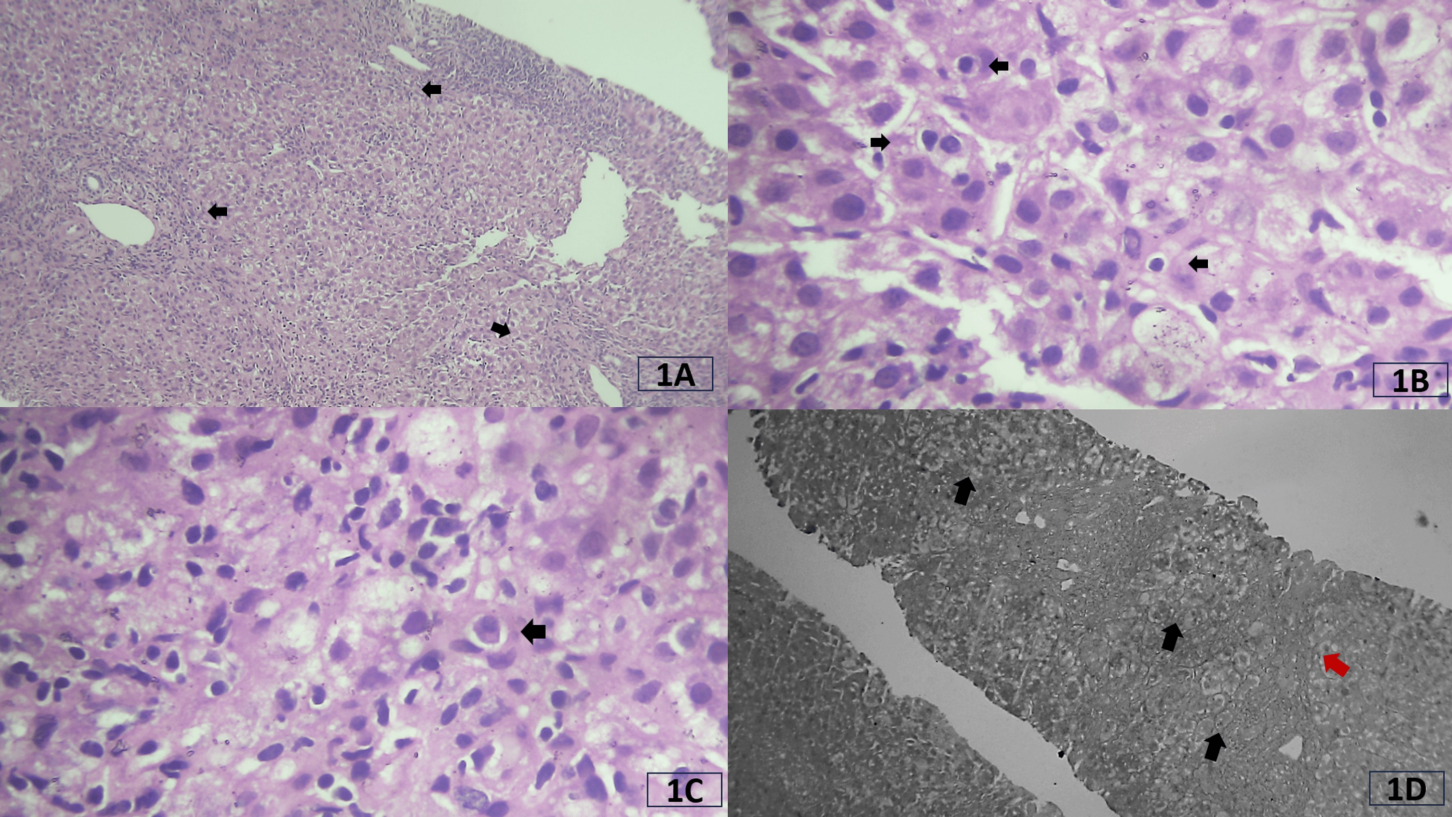
Histological features seen in liver biopsy of case 1. (A) Intermediate power view of the hematoxylin and eosin (H&E) stained section shows three portal tracts highlighted by arrows. They show moderate to marked inflammation with interface hepatitis. (B) High-power view of the H&E-stained section shows prominent emperipolesis as highlighted by the arrows. Lymphocytes are seen as dark rounded structures surrounded by a small halo in the cytoplasm of the hepatocytes. Ballooning degeneration is also evident as rounded borders with rarefied cytoplasm. (C) A High-power view of the H&E-stained section shows prominent lobular inflammation with the presence of plasma cells as highlighted by the arrow. (D) Reticulin stain on intermediate power shows disarray of normal architecture with pseudo-acinar transformation as highlighted by black arrows. There is focal bridging necrosis as highlighted by the red arrow.

A 19-year-old woman presented with a complaint of fever and yellowish discoloration of the eyes for one month. There was no history of pruritus, clay-colored stools, hematemesis, melena, or abdominal distension. She had a history of jaundice at the ages of 3 and 15 years, as well as annual episodes of jaundice for the past four years. There was no history of long-term medication use, including antitubercular treatment (ATT), over-the-counter (OTC) drugs, or herbal medications. The patient also denied a history of blood transfusions, alcohol intake, neuropsychiatric symptoms, high-risk sexual behavior, surgical procedures, or tattooing. A family history revealed that one of her maternal aunts had recurrent jaundice, and another died from an unspecified liver disease at the age of 20 years. On clinical examination, the patient exhibited icterus. Her abdomen was soft and non-tender, with no evidence of hepatosplenomegaly. Laboratory investigations are summarized in Table 1. Serological markers for hepatitis A, B, C, and E viruses were negative. Anti-liver cytosol antigen type 1 (anti-LC1) antibody was positive. Liver biopsy revealed morphological features consistent with AIH (Ishak Modified Histological Activity Index grading: 13/18) (Figure 1, Table 2). The patient was started on oral prednisolone 40 mg once daily, which was gradually tapered. She achieved good clinical and biochemical recovery over the next three to four months.

**Table 1:** Clinical, biochemical, and immunological features and treatment of AIH cases. AST: aspartate transaminase, ALT: alanine transaminase, ALP: alkaline phosphatase, HBsAg: hepatitis B surface antigen, anti-HCV: anti-hepatitis C virus antibody, anti-HAV: anti-hepatitis A virus antibody, anti-HEV: anti-hepatitis E virus antibody, ANA: antineutrophil antibody, SMA: smooth muscle antibodies, anti-LKM: anti-liver-kidney-microsome antibody, anti-LC1: anti-liver cytosol type 1 antibody, AMA: antimitochondrial antibody.

Features	Case 1	Case 2	Case 3	Case 4	Case 5
Age	19 years	60 years	21 years	21 years	75 years
Sex	Female	Female	Female	Female	Male
Clinical features					
Fever	1 month	4 months	2.5 months	9 months	No fever
Jaundice	Recurrent episodes since the age of 3 years	15 days	2 months	No	2 months
Ascites	Absent	Moderate to severe	Mild to moderate	Moderate	Present
Splenomegaly	Absent	Present	Present	Present	Present
Encephalopathy	Absent	Present	Absent	Absent	Absent
Liver function tests					
Total bilirubin	15.42	4.57	20.66	2.1	3.5
Direct bilirubin	9.27	0.94	16.93	0.7	1.5
AST	259.9	137	184.4	11.8	66
ALT	248.2	41	121.8	11.4	94
ALP	168	348	216	109	114
Total protein serum albumin	8.8	7.7	7.16	7.21	6.2
Serum albumin	3.4	2.4	2.44	3.32	2.8
Radiological evidence of chronic liver disease	No	Yes	Yes	Yes	Yes
Serology for viral hepatitis (HBsAg/anti-HCV/anti-HAV/anti-HEV)	Negative	Negative	Negative	Negative	Negative
Kayser-Fleischer (K-F) ring	Absent	Absent	Absent	Absent	Absent
AIH antibodies					
ANA	Negative	1:1000 (+++)-Cytoplasmic speckled	1:320 (++)- Speckled	1:160 (+) Cytoplasmic	Negative
SMA	Negative	Negative	Negative	Negative	Negative
Anti-LKM1	Negative	Negative	Negative	Negative	Negative
Anti-LC1	Positive	Negative	Negative	Negative	Positive
AMA	Negative	Positive	Negative	Positive	Negative
Other autoantibodies	-	-	Anti-SLA + DCT IgG- 2+	-	-
Treatment					
Steroid	Started	Started	Started	Started	Started
Azathioprine	Not started	Not started	Not started	Started	Not started
Outcome	Remission	Death	Remission	Remission	Death

**Table 2:** Summary of histomorphological characteristics of liver biopsy. HAI: histological activity index.

Case No.	HAI score (Ishaks)	Stage (Ishaks)	Portal inflammation	Interface hepatitis	Lobular activity/10x field	Confluent necrosis	Inflammatory cells	Emperipolesis	Pseudo-acinar transformation
1	13	2	3 (moderate to marked in all tracts)	3 (continuous in > 50% tracts)	3 (5 to 10)	4 (zone 3 necrosis with bridging)	Lymphocytes, plasma cells, neutrophils, eosinophils	Seen	Seen
3	4	2	1 (mild to moderate in a few tracts)	1 (focal in a few tracts)	2 (2 to 4)	0 (not seen)	Lymphocytes	Seen	Not seen
4	8	3	1 (mild in a few tracts)	0 (not seen)	1 (up to 2)	6 (panacinar and multiacinar necrosis)	Lymphocytes, plasma cells, neutrophils.	Seen	Seen
5	9	5	2 (moderate in some tracts)	3 (continuous in > 50% tracts)	3 (5 to 10)	1 (isolated confluent necrosis)	Lymphocytes, plasma cells, neutrophils.	Seen	Seen

Case 2

**Figure 2:**
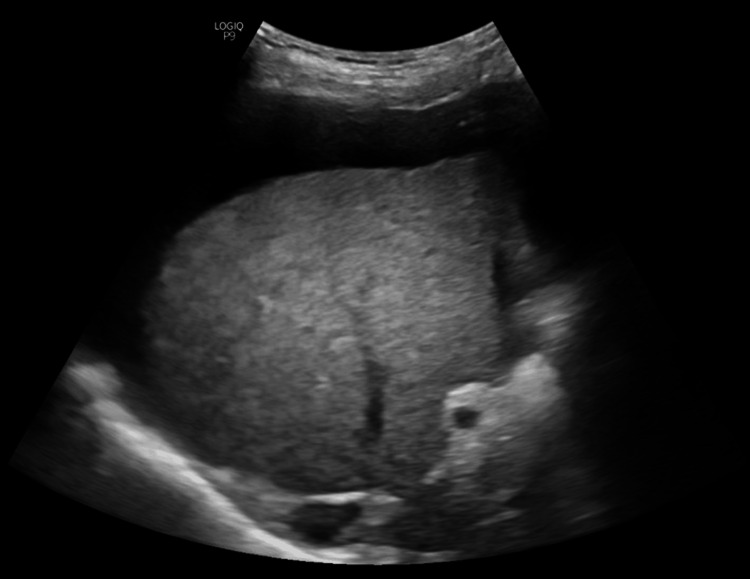
Abdominal ultrasound image of case 2. A coarsened echotexture of the liver with an irregular surface and ascites.

A 60-year-old woman presented with complaints of insidious onset, gradually progressive, painless edema of the feet for six months, and low-grade fever for four months, not associated with cough, dysuria, abdominal pain, diarrhea, or weight loss. The patient noticed jaundice and gradually progressive, painless abdominal distension for 15 days. She had a history of melena three months ago but no history of hematemesis or bleeding from other sites. Relatives reported that she had become less active, with a day-night reversal of her sleep-wake cycle. There was no history of long-term medication use (such as ATT, OTC drugs, or herbal medication), blood transfusion, or alcohol intake. On examination, she was somnolent, and asterixis (flapping tremors) was evident on both hands upon dorsiflexion of outstretched wrists. No focal neurological deficits were noted. Chest auscultation was unremarkable. An abdominal ultrasound (USG) showed features of cirrhosis and portal hypertension, including coarsened liver echotexture with an irregular surface, a dilated portal vein, splenic collaterals, and ascites (Figure 2). Ascitic fluid analysis revealed low serum ascites albumin gradient (SAAG) ascites, and cartridge-based nucleic acid amplification test (CBNAAT) for *Mycobacterium tuberculosis* was positive. Serum antinuclear antibody (ANA) and antimitochondrial antibodies (AMA) were positive. Liver biopsy could not be performed due to prolonged prothrombin time, and transjugular liver biopsy was not feasible at our center. She was diagnosed with AIH, chronic liver disease (Child-Pugh class C), portal hypertension, and tubercular ascites. She was started on a modified hepatosafe ATT regimen (ethambutol, levofloxacin, streptomycin), oral steroids, and treatment for complications of liver cirrhosis.

One month after discharge, the patient returned with drowsiness and decreased urine output. She was admitted and managed for hepatic encephalopathy and renal failure but showed no improvement and succumbed to her illnesses.

Case 3

**Figure 3:**
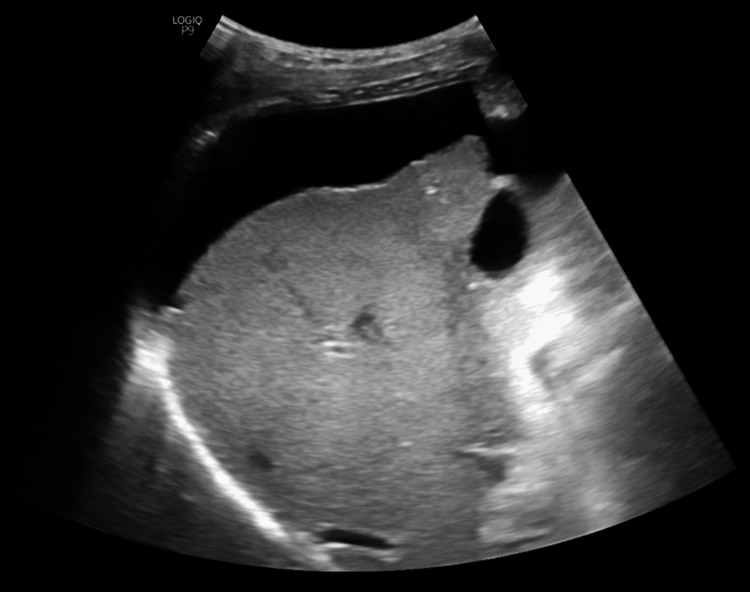
USG abdomen of case 3. Ultrasound (USG) image reveals a coarsened echotexture of the liver with an irregular surface and ascites.

**Figure 4:**
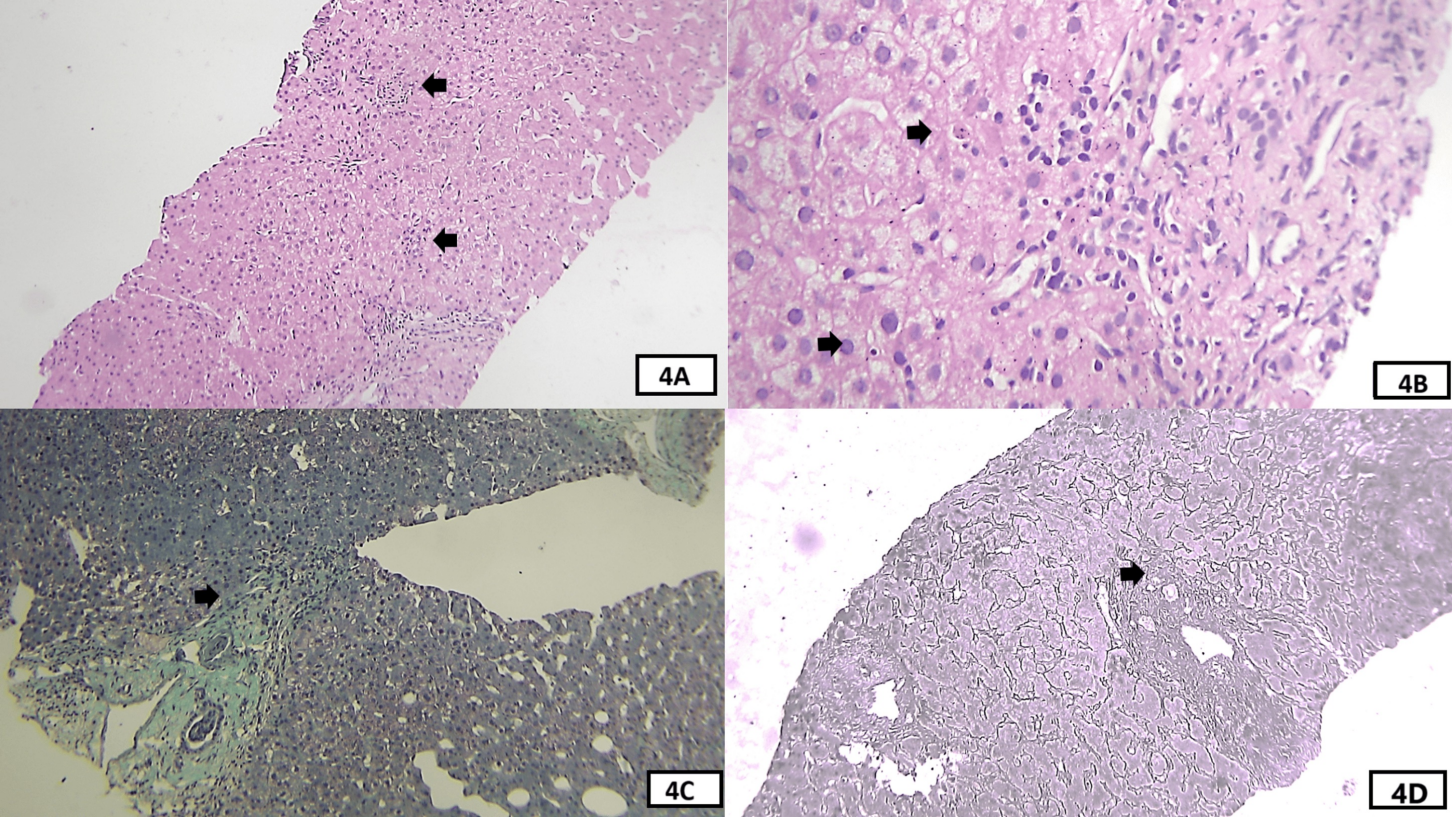
Histological features seen in liver biopsy of case 3. (A) An intermediate-power view of the H&E-stained section of the liver biopsy shows tiny foci of lobular inflammation, highlighted by black arrows. The portal tract with mild to moderate inflammation is seen in the lower part. (B) A high-power view of the H&E-stained section shows the portal tract on the right side with interface hepatitis. The upper black arrow shows apoptotic hepatocytes and the lower arrow shows emperipolesis. (C) Intermediate-power view of the Masson trichrome stain shows a portal tract in the lower left corner with a green color. The arrow highlights focal interface hepatitis. The portal tract is mildly expanded. Hepatocytes appear reddish. (D) An intermediate-power view of the reticulin stain section shows predominantly intact architecture. There is focal interface hepatitis as highlighted by the arrow as a disturbance in the continuity of the limiting plate.

A 21-year-old woman presented with fever for 2.5 months, yellowish discoloration of the eyes for two months, and abdominal distension for two weeks. Clinical examination revealed icterus, ascites, and splenomegaly. There was no history of predisposing factors for acute or chronic liver disease (as mentioned in previous cases). Serological markers for hepatitis A, hepatitis B, hepatitis E, and hepatitis C were negative. An abdominal USG showed coarsened liver echotexture with an irregular surface, a dilated portal vein, and ascites (Figure 3). Serum ANA was positive at a 1:320 titer with a speckled pattern. Percutaneous liver biopsy revealed features of chronic active hepatitis (Figure 4, Table 2). She was diagnosed with AIH, chronic liver disease (Child-Pugh class B), and portal hypertension. Treatment with tablet prednisolone was initiated, and the patient was followed up monthly. Over three months, her liver function test results normalized, and ascites resolved. Steroids were gradually tapered to 5 mg once daily, and she was maintained on this dose.

Case 4

A 21-year-old woman presented with complaints of menorrhagia. During the evaluation, anemia and thrombocytopenia were detected. One month later, she developed insidious onset and gradually progressive breathlessness (mMRC grade III) due to bilateral exudative pleural effusion. She visited a physician and was prescribed antitubercular therapy (ATT), although there was no microbiological evidence of tuberculosis. Three months later, she developed ascites and pedal edema and presented to us. There was no history of fever, arthritis, malar rash, photosensitivity, oral ulcers, alopecia, or Raynaud’s phenomena. Additionally, she reported no gastrointestinal bleeding, altered sensorium, decreased urine output, or predisposing factors for liver injury.

**Figure 5:**
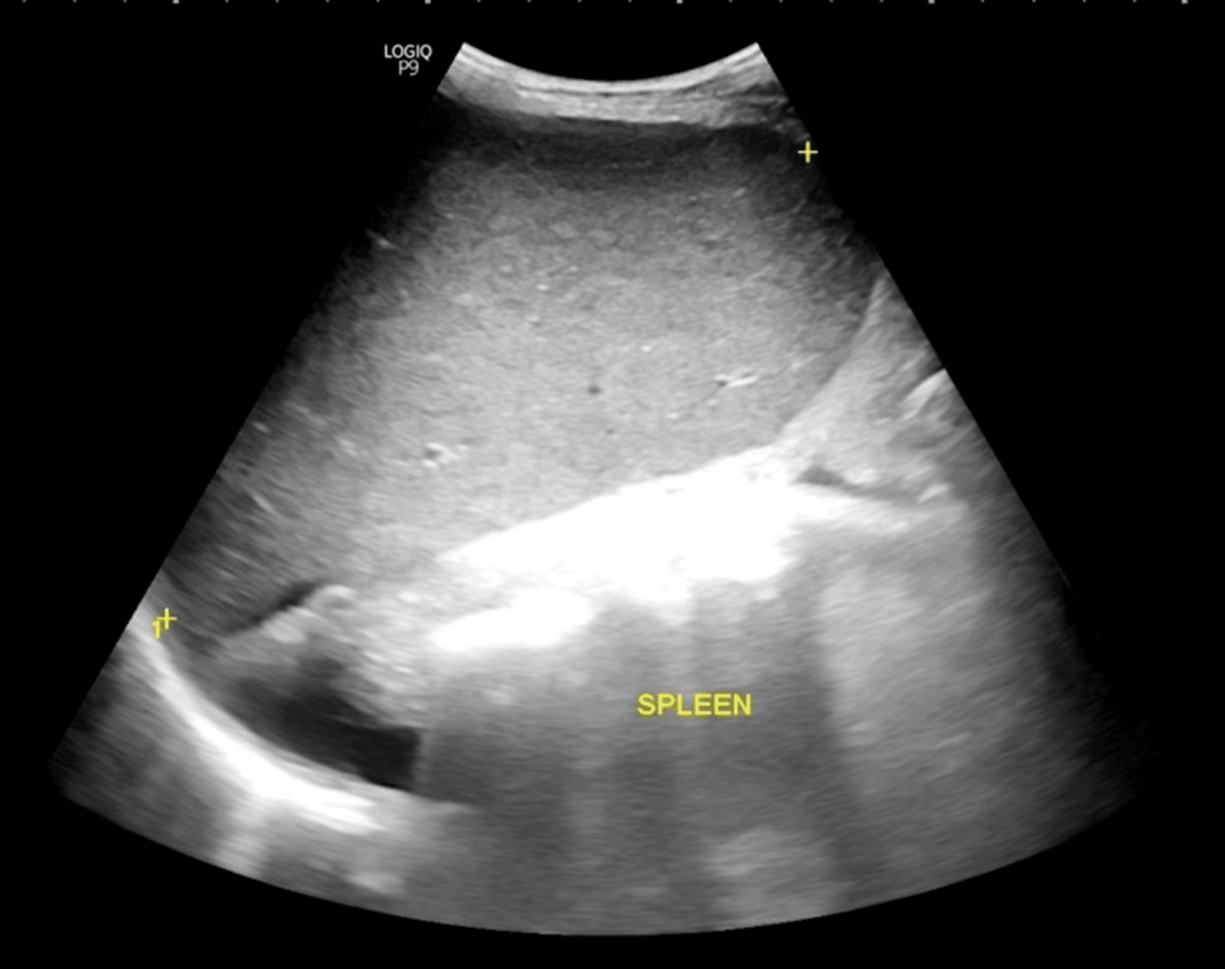
Ultrasound abdomen image of case 4. The image shows mild hepatomegaly and splenomegaly.

**Figure 6:**
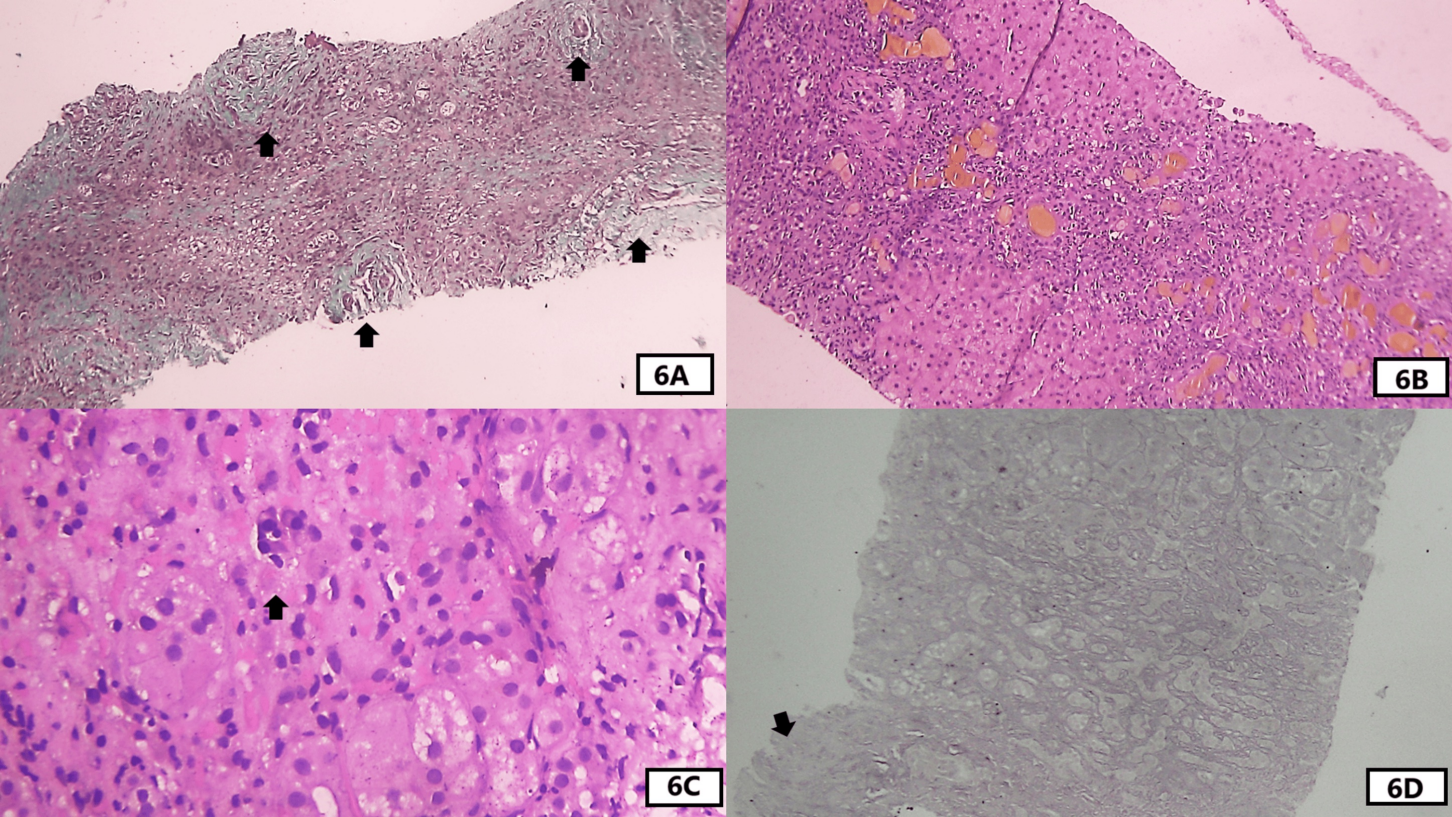
Histological features seen in liver biopsy of case 4. (A) An intermediate-power view of Masson trichrome stain from one core shows portal tracts coming close to each other as highlighted by arrows. This is because of extensive loss of the liver parenchyma owing to panacinar necrosis. (B): An intermediate-power view of the H&E section from the other core shows extensive bridging necrosis with necrotic septa rich in inflammatory cells. They also contain many bile ductuli with bile plugs. Viable hepatocytes are seen above and below the septa as bright pink areas. (C) A high-power view of the H&E-stained section shows focal plasma cell aggregates in the parenchyma with ballooning of a few hepatocytes. (D)  Intermediate-power of the reticulin-stained section shows disarray with acinar transformation. The middle part shows extensive collapse with a few viable and proliferating hepatocytes forming elongated structures. The lowermost part shows part of the portal tract.

A complete blood count revealed pancytopenia. ANA was positive with a titer of 1:160. USG abdomen showed mild hepatomegaly, splenomegaly, and mild to moderate ascites (Figure 5). Serum C3 and C4 levels were low, and the direct Coombs test was positive. Serum anti-soluble liver antigen (SLA) was also positive. Liver biopsy revealed histological features consistent with AIH (Figure 6, Table 2). The patient was diagnosed with systemic lupus erythematosus (SLE), AIH, cirrhosis of the liver (Child-Pugh class B), and portal hypertension. She was started on hydroxychloroquine, azathioprine, and gradually tapering doses of prednisolone. On follow-up, the patient reported significant improvement, with resolution of ascites and pleural effusions, and normalization of blood counts. She is currently doing well on prednisolone 5 mg and azathioprine 50 mg per day.

Case 5

**Figure 7:**
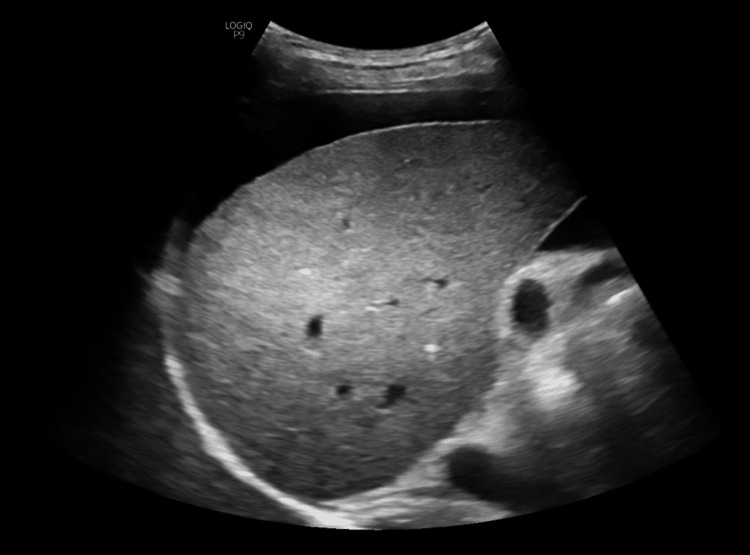
Ultrasound abdomen of case 5. The image reveals moderate ascites, a shrunken liver with coarsened echotexture, and an irregular surface.

**Figure 8:**
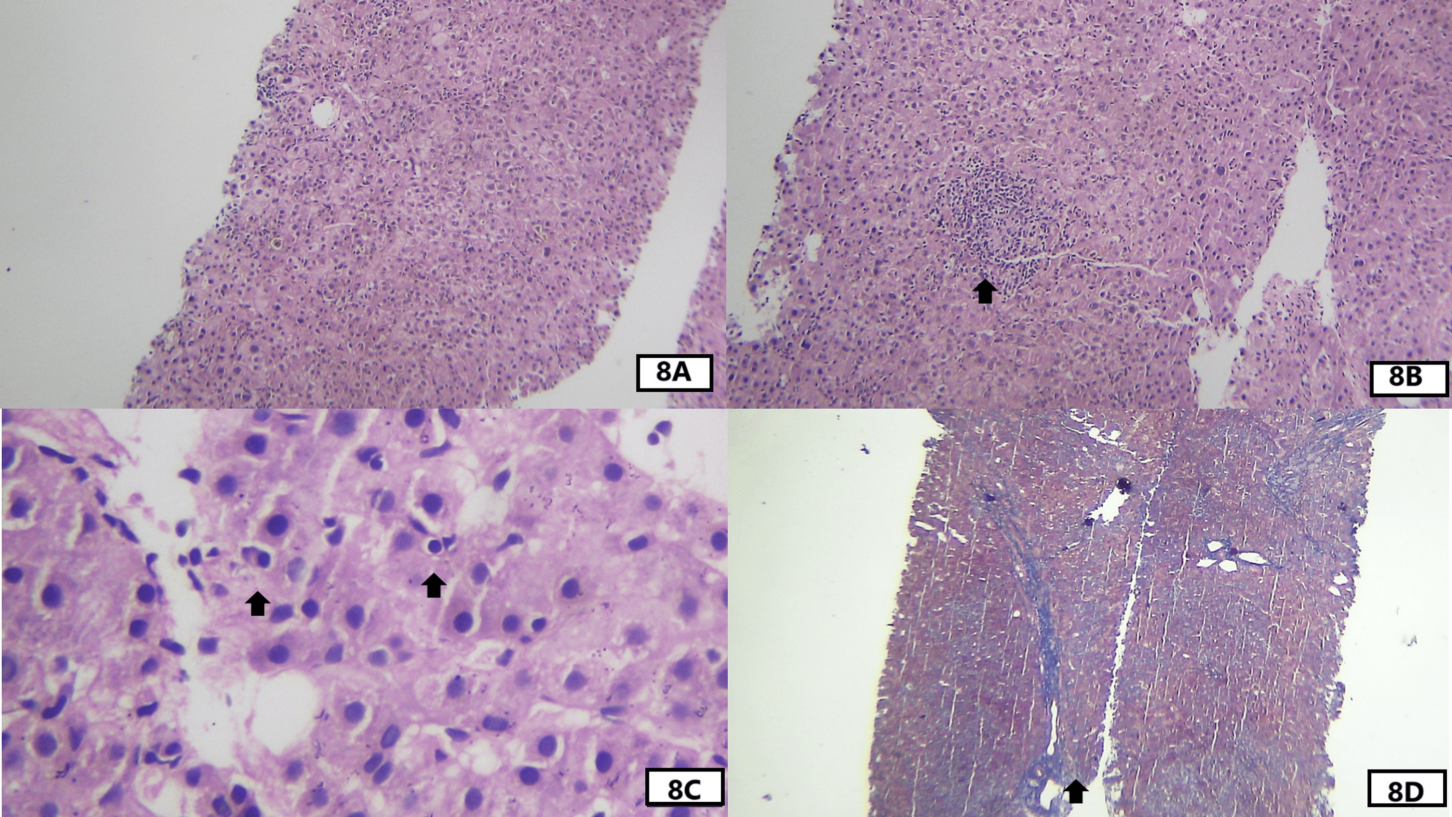
Histological features seen in liver biopsy of case 5. (A) An intermediate-power view of the H&E-stained section shows prominent lobular activity. (B) An intermediate-power view of the H&E-stained section from another core focuses on confluent necrosis with prominent inflammation. (C) A high-power view shows emperipolesis on the right side and plasma cells on the left side as highlighted by the arrow. (D) An intermediate-power view of the Masson trichrome stain shows rounded collagen septa (blue color) enclosing an incomplete nodule. Other parts show frequent bridging fibrosis.

A 75-year-old man presented to our emergency department with abdominal distension and jaundice for two months. He reported anorexia and approximately 9 kg of weight loss. There was no history of hematemesis, melena, altered sensorium, decreased urine output, or fever. Ascitic fluid analysis revealed high SAAG ascites, suggestive of portal hypertension. USG abdominal showed moderate ascites, a dilated portal vein (12 mm), a shrunken liver with coarsened echotexture, and an irregular surface (Figure 7). The patient was diagnosed with acute-on-chronic liver failure and portal hypertension. There was no history of alcohol consumption, viral markers for hepatitis B and C were negative, and no Kayser-Fleischer ring was observed. ANA was negative, and anti-liver cytosol antigen type 1 (LC1) antibody was positive. Liver biopsy revealed histological findings consistent with AIH (Figure 8, Table 2). The patient was started on prednisolone 40 mg once daily along with a diuretic and beta-blocker. On follow-up, his bilirubin levels decreased slightly, but he developed hepatic encephalopathy. Three months later, he succumbed to his illness.

## Discussion

AIH, a chronic disease characterized by hepatocellular necrosis and inflammation, is considered rare in the Indian population. In two Indian hospital-based cohort studies, the proportions of AIH patients among those presenting with acute or chronic liver diseases were 1.3% and 1.58% [3,4]. In Europe, the prevalence ranges between 16 and 18 cases per 100,000 population [5]. AIH can affect individuals of all age groups, with a female preponderance. For example, in a Swedish study, 76% of AIH patients were women [6], while in an Indian study, the proportion of female AIH patients was 64.8% [3]. In our case series, three cases involved young adults, and two involved older adults (>60 years). Four out of the five cases were women. A bimodal age distribution at presentation has been reported in various studies, with one peak during childhood or adolescence and another in middle age, between the 4th and 6th decades of life [5].

The symptoms of AIH are nonspecific, and patients can present with variable complaints ranging from asymptomatic biochemical abnormalities in liver function tests to fatigue, jaundice, features of chronic liver disease, or acute fulminant hepatitis. The acute presentation of AIH may represent two distinct entities: acute exacerbation of chronic AIH or acute AIH without underlying chronic changes [5]. Cirrhosis is present in 28%-33% of adults at diagnosis, particularly in older adults, and in 38% of children [1]. One of our patients (case 1) presented with recurrent hepatitis without evidence of chronic liver disease. The other four patients exhibited clinical features of decompensated cirrhosis. Fever (present in 4 of 5 cases) and jaundice (present in 4 of 5 cases) were the most common symptoms in our series. In a study by Choudhuri et al., fever was present in 21% of subjects, with 50% presenting with chronic hepatitis, 34.2% with cirrhosis, and 13.1% with acute hepatitis [4].

In our series, three young (younger than 30 years) female patients were diagnosed with AIH. In two of these three cases, cirrhosis had already developed at the time of diagnosis, suggesting a prolonged asymptomatic period and the progressive nature of hepatic injury in AIH.

In two patients (cases 2 and 4), AMA was positive, which is typically seen in primary biliary cirrhosis (PBC) or in cases of overlap between AIH and PBC. A liver biopsy is recommended in such cases to confirm the diagnosis. In case 2, prothrombin time was prolonged. Although liver biopsy can be performed via a transjugular or transfemoral approach in patients with coagulopathy, this facility was unavailable at our center. In case 4, we performed a liver biopsy but did not find any features of PBC. Notably, AMA may be present in certain patients with AIH even in the absence of histological features of bile duct injury or loss [7]. In case 2, a positive CBNAAT for *Mycobacterium tuberculosis *explained the low serum ascites albumin gradient (SAAG <1.1) ascites. Modified ATT was initiated. Evidence suggests that tuberculosis is 15 times more common in patients with cirrhosis than in the general population [8]. An acquired immunodeficient state due to cirrhosis likely contributes to this increased susceptibility.

Other autoimmune disorders such as rheumatoid arthritis, SLE, vitiligo, and juvenile diabetes mellitus may be seen in association with AIH diagnosis. One of our patients (case 4) was diagnosed with SLE. The other four cases did not have coexistent autoimmune disorders.

AIH lacks a specific diagnostic marker; thus, the diagnosis of AIH requires a combination of clinical and biochemical features (elevated serum aspartate transaminase (AST) and alanine aminotransaminase (ALT) levels and increased serum IgG concentration), characteristic histological changes, and the presence of characteristic autoantibodies [9]. We considered AIH in our cases after excluding common causes of liver injury (e.g., alcohol, viral hepatitis, drug-induced liver injury, Wilson’s disease). AIH is classified as AIH type I and type II based on the presence of autoantibodies. Type I AIH is associated with ANA, AMA-M2, and anti-smooth muscle cell antibodies. It is more common in young women in North America and Northern Europe. Type II AIH is more common in the Mediterranean population and is not associated with ANA but with anti-liver/kidney microsomal (LKM) or anti-LC1 antibodies [10]. Three patients were diagnosed with type I AIH and two patients with type II AIH. Type I AIH patients were all women and had chronic symptoms. Type II AIH can affect people of all ages but is more common in the age group 2-14 years [8]. One of our type II AIH patients was a young woman who presented with recurrent episodes of acute hepatitis. Another patient with type II AIH was an older man who presented with cirrhosis. It is characterized by anti-LKM type 1 (anti-LKM1) and an absence of anti-actin, anti-mitochondria, and anti-nucleus antibodies [11]. Acute presentation is more common, and relapse rates are higher in this subset of AIH [1].

Biochemical tests of liver function in AIH are often abnormal but do not always correlate with the severity of the illness or the histological stage. Some AIH patients may present with normal serum bilirubin, alkaline phosphatase (ALP), and globulin levels despite slight AST/ALT elevation. In a subset of AIH patients with markedly elevated ALP levels, clinical and laboratory features may overlap with those of PBC.

Liver biopsy shows interface hepatitis or piecemeal necrosis, that is, a mononuclear cell infiltrate, and may have plasma cells. Necroinflammatory activity along with regenerative nodules is reflected by “rosette” formation, regenerative “pseudolobule”, and emperipolesis. Septal fibrosis, bridging fibrosis, and cirrhosis are common. Bile duct injury and granulomas are seen in a subgroup of patients with AIH having features overlapping those of PBC. We used the Ishak scoring system for the histological assessment of liver biopsy. This helps ascertain the stage (based on fibrosis) and grade (based on necroinflammation) of liver pathology [12]. The histological features of the four cases are summarized in Table 2.

Most AIH patients respond well to steroid treatment, and serum transaminases improve to levels within the normal range [5]. Either of the two regimens can be used for induction: a prednisolone-only (40-60 mg/day in adults; 1-2 mg/kg in children) regimen or a low-dose prednisolone (20-40 mg/day) with azathioprine (adult dose 50-150 mg/day) [1]. After induction, prolonged maintenance therapy with a very low dose of prednisolone (5-10 mg/day) is continued. Second-line immunosuppressants such as mycophenolate mofetil, cyclosporine, tacrolimus, rituximab, and infliximab have also been used in the management of different scenarios such as intolerance to first-line drugs, incomplete response, or treatment failure.

All the patients were initiated on steroid therapy. Three patients (all women) responded well to the immunosuppressant therapy, achieving normal biochemical parameters over three to four months. They are currently doing well on prednisolone 5 mg/day. Two female patients (cases 1 and 3) did not accept treatment with azathioprine (AZA) owing to the possible risk of teratogenicity. However, case 4 tolerated AZA very well. It is recommended to attempt withdrawal of immunosuppressant therapy at least 48 months after sustained normal serum levels of AST, ALT, and IgG [5]. After drug withdrawal, 50%-87% of adults and 60%-80% of children may relapse. A liver transplant is the only option for patients who do not show a good response to steroid therapy and who present with acute or fulminant hepatic failure.

Based on some estimates of the natural history of AIH, the 10-year survival for treated AIH cases is 80−98% and that for untreated patients is 67%. Cirrhosis is associated with reduced survival [1]. Two of our patients who died were older adults with advanced cirrhosis.

## Conclusions

AIH is a distinct liver disease with variable presentations. It exhibits a bimodal age distribution and is more common in women. While AIH can coexist with other liver diseases such as PBC and viral hepatitis, it should be suspected in patients presenting with features of acute or chronic liver disease, especially when common etiologies have been excluded. The presence of autoantibodies (ANA, ASMA, anti-LKM1/3, SLA, anti-LC1) and characteristic histological features on liver biopsy are pivotal for diagnosing AIH. Immunosuppression with steroids combined with azathioprine is the first-line therapy for inducing remission, followed by prolonged maintenance therapy with low-dose steroids. Advanced age at diagnosis and the presence of cirrhosis are associated with poorer outcomes. Thus, early diagnosis and timely treatment are crucial to achieving remission and preventing progression to end-stage liver disease.
